# Solar-microbial hybrid device based on oxygen-deficient niobium pentoxide anodes for sustainable hydrogen production†
†Electronic supplementary information (ESI) available: Video of the generations of gas by the hybrid device under illumination. See DOI: 10.1039/c5sc03249k



**DOI:** 10.1039/c5sc03249k

**Published:** 2015-09-18

**Authors:** Mingyang Li, Xinjun He, Yinxiang Zeng, Meiqiong Chen, Ziyang Zhang, Hao Yang, Pingping Fang, Xihong Lu, Yexiang Tong

**Affiliations:** a KLGHEI of Environment and Energy Chemistry , MOE of the Key Laboratory of Bioinorganic and Synthetic Chemistry , School of Chemistry and Chemical Engineering , Sun Yat-Sen University , Guangzhou 510275 , China . Email: fangpp3@mail.sysu.edu.cn ; Email: luxh6@mail.sysu.edu.cn ; Email: chedhx@mail.sysu.edu.cn; b Dongguan Key Laboratory of Green Energy , City College of Dongguan University of Technology , Wenchang Road , Dongguan , 523419 China

## Abstract

Hydrogen gas is emerging as an attractive fuel with high energy density for the direction of energy resources in the future. Designing integrated devices based on a photoelectrochemical (PEC) cell and a microbial fuel cell (MFC) represents a promising strategy to produce hydrogen fuel at a low price. In this work, we demonstrate a new solar-microbial (PEC–MFC) hybrid device based on the oxygen-deficient Nb_2_O_5_ nanoporous (Nb_2_O_5–*x*_ NPs) anodes for sustainable hydrogen generation without external bias for the first time. Owing to the improved conductivity and porous structure, the as-prepared Nb_2_O_5–*x*_ NPs film yields a remarkable photocurrent density of 0.9 mA cm^–2^ at 0.6 V (*vs.* SCE) in 1 M KOH aqueous solution under light irradiation, and can achieve a maximum power density of 1196 mW m^–2^ when used as an anode in a MFC device. More importantly, a solar-microbial hybrid system by combining a PEC cell with a MFC is designed, in which the Nb_2_O_5–*x*_ NPs electrodes function as both anodes. The as-fabricated PEC–MFC hybrid device can simultaneously realize electricity and hydrogen using organic matter and solar light at zero external bias. This novel design and attempt might provide guidance for other materials to convert and store energy.

## Abstract 2

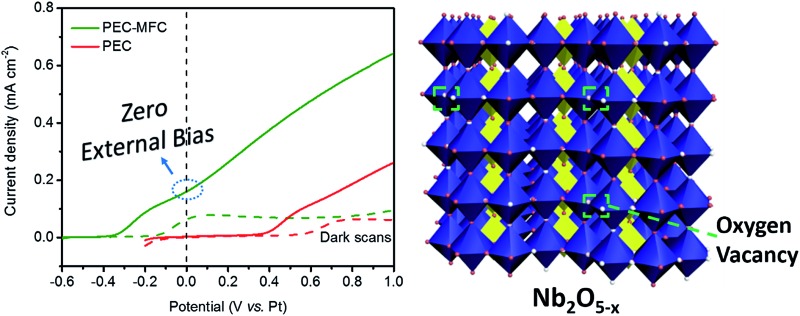
A new solar-microbial hybrid device based on oxygen-deficient Nb_2_O_5_ anodes for sustainable hydrogen generation without external bias was demonstrated.

## 1. Introduction

The rational utilization of renewable and clean energy has drawn more and more attention due to the increasing energy demands and ever-growing environmental concerns. Producing hydrogen by photoelectrochemical (PEC) water splitting over a semiconductor photoelectrode is emerging as the most promising way that can directly convert solar energy into clean hydrogen.^1–3^ Hydrogen from water splitting is known to be a thermodynamically uphill or endothermic process and can be described as the following equation:

1 H_2_O(l) → H_2_(g) + 1/2O_2_(g), *E*^0^ = 1.23 V *vs.* NHE

To drive this reaction, a minimal energy of 237.2 kJ mol^–1^ that is equal to a potential of 1.23 V *vs.* NHE is required.^2,3^ In this respect, it requires the conduction band (CB) and valence band (VB) edges of the photoelectrode to straddle the reduction and oxidation potentials of water. Specifically, *E*_CB_ should be above *E*_red_ (H_2_/H^+^) and *E*_VB_ should be below *E*_ox_ (OH^–^/O_2_). Besides having suitable band edge positions, an ideal photoelectrode material for efficient solar water splitting should also possess strong light-harvesting ability, good chemical stability, fast charge transport and low cost.^4–6^ However, most of the developed photoelectrodes cannot satisfy these requirements simultaneously. On the other hand, an external bias is always needed for practical applications to improve their charge separation and/or overcome an overpotential for the low lying CB edge, which leads to increased cost of hydrogen production.^7–10^ In order to address the above-mentioned energy loss, numerous efforts have been devoted to designing nanostructured photoelectrodes and catalysts as well as optimizing the PEC configuration.^11–13^ Nevertheless, the current efficiency of PEC water splitting is still not satisfactory for its high energy demand.^14,15^ Alternatively, the energy required for water splitting obtained from a renewable energy source is a more cost-effective approach to generate hydrogen.

Microbial fuel cells (MFCs) are of great interest since they can directly produce electricity from organic waste and biomass at a low cost. MFCs are bioelectrochemical devices that can convert chemical energy stored in organic matter into electricity with the help of microorganisms.^16–23^ This feature makes them very attractive as a green energy supply to overcome the thermodynamic constraints and compensate for the energy loss of PEC water splitting. Recently, Wang *et al.* demonstrated that the hydrogen gas could be continuously produced based on solar light and biomass recycling through coupling PEC water splitting with a TiO_2_ nanowire-arrayed photoanode and microbial electrohydrogenesis with *Shewanella oneidensis* MR-1 in a PEC–MFC hybrid device.^16^ Despite these achievements, the unsatisfactory performances of the current MFCs and PEC cells are the main obstacles for their practical applications. The electrode material is the most key factor that determines the performances of both the MFCs and PEC cells.^16–19,24–27^ Therefore, the exploration and development of an advanced electrode material for improving the efficiency of PEC cells and the output power density of the MFCs are highly desirable.

In this work, we demonstrate the feasibility of oxygen-deficient Nb_2_O_5_ nanoporous (Nb_2_O_5–*x*_ NPs) films as a high-performance anode material for both the PEC cells and MFCs. Nb_2_O_5_ is one of the most important n-type semiconductor materials for dye-sensitized solar cells and photocatalysts in terms of its excellent photoactivity, non-toxicity and environmental friendliness.^28–33^ It has a similar bandgap to TiO_2_ and ZnO, and favourable band-edge positions that can straddle the redox potential of water photoelectrolysis. Moreover, recent reports have shown that Nb_2_O_5_ possesses better biocompatibility than TiO_2_ and higher stability than ZnO (amphoteric oxide).^34,35^ All of these characteristic features make it a very promising photoanode and anode for PEC cells and MFCs, respectively. However, Nb_2_O_5_ suffers from poor electrical conductivity, which seriously limits its wide applications. Herein, we developed a facile approach to significantly boost the conductivity of Nb_2_O_5_ by creating oxygen vacancies. Nb_2_O_5–*x*_ NPs films were readily obtained by anodic oxidation of Nb foil and hydrogenation treatment. Benefiting from the appropriate bandgap, suitable band levels, improved conductivity and good biocompatibility, the Nb_2_O_5–*x*_ NPs films exhibited superior performances in both the PEC cells and MFCs. The Nb_2_O_5–*x*_ photoanode achieved a remarkable photocurrent density of 0.9 mA cm^–2^ at 0.6 V (*vs.* SCE) in 1 M KOH aqueous solution, and the MFCs with the Nb_2_O_5–*x*_ NPs anodes (denoted as Nb_2_O_5–*x*_-MFCs) exhibited a maximum power density of 1196 mW m^–2^. More interestingly, a PEC–MFC hybrid device, by interfacing a Nb_2_O_5–*x*_-based PEC device and a Nb_2_O_5–*x*_-based MFC device, was designed and continuous hydrogen gas could be produced at zero external bias by biodegradable organic matter and solar light.

## 2. Experimental section

### 2.1 Fabrication of Nb_2_O_5_ and Nb_2_O_5–*x*_ NPs films

Nb_2_O_5_ NPs films were synthesized by the simple electrochemical anodization of Nb films. Before the anodization, the Nb film was washed by ultrasonication in acetone, ethanol and then deionized water successively. The Nb film was anodized at an applied potential of 20 V for 30 min at room temperature, with the Nb film as the working electrode and a graphite rod of about 4.0 cm^2^ as the cathode in a glycerol aqueous solution (90 vol% glycerol : 10 vol% H_2_O) containing 1.5% NH_4_F. The as-prepared Nb_2_O_5_ NPs films were cleaned with ethanol and DI water, and then were annealed at 450 °C for 60 min in air to enhance the mechanical stability and crystallinity. Finally, the Nb_2_O_5–*x*_ NPs films were obtained by annealing the Nb_2_O_5_ NPs films in a hydrogen atmosphere at various temperatures ranging from 450–550 °C for 60 min. Thermal treatment was performed in a home-built tube furnace filled with ultrahigh purity hydrogen gas.

### 2.2 Fabrication of MFC

At first, *E. coli* K-12 (ATCC 25922) was cultured at 37 °C in a fertile medium (a mixture of 10.0 peptone, 5.0 g NaCl and 3.0 g beef powder per liter) in an anaerobic test tube, which was sterilized at 121 °C in an autoclave for 20 min in advance. Secondly, 10 mL of the *E. coli* K-12 culture medium was inoculated into the nitrogen saturated anolyte, after growing for 18 h in the incubator. A cubic MFC with a single chamber (4 cm × 5 cm × 5 cm) was used in this work, which consists of a polymethyl methacrylate chamber and a membrane cathode assembly on one side (4 cm × 4 cm). The sample electrodes (4 cm × 4 cm) were directly used as the anodes. The cathode was prepared by hot-pressing carbon paper on one side of a cation exchange membrane (CEM). The carbon paper cathode was pasted with 12.5 mg cm^–3^ commercial Pt-catalyst (40 wt% Pt/C) in a mixture of Nafion (5%). Phosphate-buffered basal medium (PBBM) with 5 mM HNQ, 10.0 g glucose and 5.0 g yeast extract per liter was used as the anolyte. The PBBM consisted of 8 g NaCl, 0.2 g KCl, 3.63 g Na_2_HPO_4_·12H_2_O, and 0.24 g KH_2_PO_4_ per litre. To remove the possible metal and biomass contamination, the cell was washed with 1 mol L^–1^ HCl and 1 mol L^–1^ NaOH and rinsed with sterile water before the inoculation.

### 2.3 Material characterization

The surface structure, morphology and composition of the samples were characterized by scanning electron microscopy (SEM, Quanta 400), X-ray diffraction (XRD, Bruker, D8 ADVANCE) with Cu Kα radiation (*λ* = 1.5418 Å), and transmission electron microscopy (TEM, JEM2010-HR). X-Ray Photoelectron Spectroscopy (XPS, ESCALab250) with 200 W Al Kα radiation was used to measure the chemical bonding state and chemical state of the samples. The C 1s peak at 284.8 eV from adventitious carbon was regarded as the energy reference. Raman spectroscopy was conducted on a Laser Micro-Raman Spectrometer (Renishaw inVia) using a visible laser (*λ* = 514.5 nm) with an output laser power of 50 mW as the excitation wavelength at room temperature. The optical properties of the products were measured with a UV-Vis-NIR spectrophotometer (UV, Shimadzu UV-3150). EPR spectra were studied on powdered products by a conventional Bruker spectrometer (Bruker, A300-10-12) operating at an X-band frequency and magnetic field modulation of 100 kHz, with a microwave power of 2.23 mW and a modulation amplitude of 8 G at 88 K. The resonance lines were simulated by the Bruker WINEPR SimFonia program.

### 2.4 Photoelectrochemical and electrochemical measurements

PEC measurements were carried out in a three-electrode cell with a flat quartz window to facilitate illumination of the photoelectrode surface. The Pt foil electrode and a saturated calomel electrode were used as the counter and reference electrode, respectively. A 1.0 M NaOH aqueous solution was used as the electrolyte, and the illumination source was a 150 W xenon lamp coupled with an AM 1.5G filter. Additionally, a 1.0 M NaOH aqueous solution with 10.0 g glucose per liter was used in the control experiment. Incident photon to current conversion efficiencies (IPCE) were collected by a CHI 760D electrochemical station with a solar simulator (Newport 69920, 1000 W xenon lamp), coupled with an infrared water filter (Oriel 6127) and aligned monochromator (Oriel Cornerstone 130 1/8 m). To quantitatively reveal the interplay between the photoactivity and light absorption, incident photon to current conversion efficiencies (IPCE) were measured on the Nb_2_O_5_ and Nb_2_O_5–*x*_ NPs photoanodes at 0.2 V *vs.* SCE. IPCE can be expressed by the equation:

2 IPCE = (1240*I*)/(*λJ*_light_)

where *I* is the measured photocurrent density at a specific wavelength, *λ* is the wavelength of incident light, and *J*_light_ is the measured irradiance at a specific wavelength. The conductivity of the as-prepared films is measured by two-point current–potential (*I*–*V*) curves by a typical two electrode system. One of the electrodes is fixed on the substrate and the other fixed on the surface of the film. All of the process is completed with silver paste and under the same conditions.

## 3. Results and discussion

### 3.1 Synthesis and characterization of Nb_2_O_5–*x*_ NPs films

**Fig. 1:**
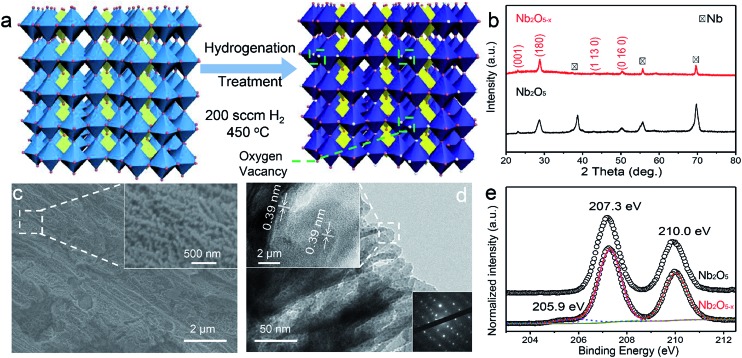
(a) Crystal structure of Nb_2_O_5_ and showing the formation of oxygen vacancies; (b) XRD spectra of Nb_2_O_5–*x*_ NPs film; (c) SEM images of Nb_2_O_5–*x*_ NPs film; inset: magnified SEM image of Nb_2_O_5–*x*_ NPs film; (d) TEM images of Nb_2_O_5–*x*_ NPs film; the upper left inset: lattice-resolved TEM image collected at the edge of the NP film; the bottom right inset: the diffraction pattern; (e) normalized Nb 3d core-level XPS spectra collected for Nb_2_O_5_ and Nb_2_O_5–*x*_ NPs films.

Nb_2_O_5–*x*_ NPs films were obtained through a two-step procedure, which involves the anodic oxidation of Nb foil and heat treatment in a hydrogen atmosphere. Firstly, the Nb_2_O_5_ NPs films were synthesized on a niobium film substrate by a simple anodic oxidation method and annealed in air at 450 °C (Experimental section). The SEM images show that the white homogeneous film obtained on the metal substrate is composed of dense nanoporous arrays with ∼50 nm diameter (Fig. S1, ESI†). To introduce oxygen vacancies into the Nb_2_O_5_ NPs films, the as-prepared Nb_2_O_5_ NPs films were then annealed in a hydrogen atmosphere for an additional 60 min at 500 °C. The synthetic process of the hydrogen treatment is illustrated in Fig. 1a. After the treatment, the film colour changed from white to blue (Fig. S2, ESI†), suggesting a possible modification in the crystal structure. To identify the possible phase transformation, XRD patterns of the Nb_2_O_5_ and Nb_2_O_5–*x*_ NPs films are shown in Fig. 1b. Sharp diffraction peaks centered at 2*θ* angles of 22.6°, 28.4° and 50.8°, corresponding to the (001), (180) and (0 16 0) planes of Nb_2_O_5_ (JCPDS #30-0873), are observed for both samples, indicating that the NPs films are well crystalline with a similar phase. SEM studies also reveal there are no obvious morphological changes for the Nb_2_O_5–*x*_ NPs film after the hydrogenation (Fig. 1c). Fig. 1d displays the TEM image and the selected-area electron diffraction (SAED) of the Nb_2_O_5–*x*_ NPs film. The TEM image obviously shows that the porous Nb_2_O_5–*x*_ sample consists of the nanoporous arrays. The clear lattice fringes with a spacing of 0.39 nm are indexed to the (001) plane of the orthorhombic Nb_2_O_5_ samples. Meanwhile, the bright and well-arranged diffraction spots further confirm the highly crystalline nature of the Nb_2_O_5–*x*_ NPs film.

**Fig. 2:**
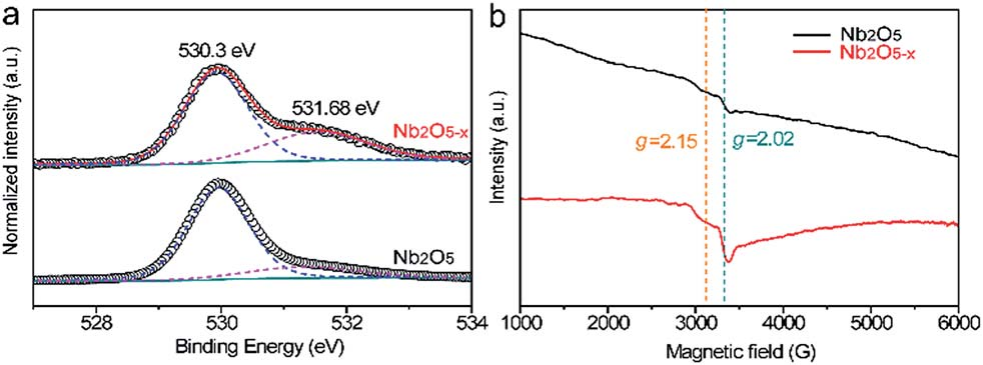
(a) Normalized O 1s core-level XPS and (b) EPR spectra collected for Nb_2_O_5_ and Nb_2_O_5–*x*_ NPs films.

The UV-visible absorption spectra of Nb_2_O_5_ and Nb_2_O_5–*x*_ NPs films were collected to investigate the influence of hydrogenation on the optical absorption (Fig. S2, ESI†). Both of the samples exhibit similar light absorption in the UV region and the Nb_2_O_5–*x*_ NPs film shows substantially higher absorption when compared to pristine Nb_2_O_5_ in the region of 400–800 nm, which suggests Nb_2_O_5–*x*_ may absorb more visible light. The band gap of the Nb_2_O_5–*x*_ NPs film is calculated to be 3.26 eV, while it is 3.35 eV for the pristine Nb_2_O_5_ NPs film, indicating the hydrogenation has a negligible effect on its band gap. To further investigate the effect of the hydrogen treatment on the chemical state of Nb_2_O_5_, XPS analysis was performed. The XPS survey of the Nb_2_O_5–*x*_ NPs film was collected to prove that no other impurities were introduced after the hydrogen treatment (Fig. S3, ESI†). Fig. 1e displays the normalized high resolution Nb 3d core level XPS spectra. Both of the samples have two peaks located at 207.3 and 210 eV, which are ascribed to the regular Nb 3d signals for Nb^5+^.^36,37^ Remarkably, the Nb_2_O_5–*x*_ NPs film displays an additional peak which emerged at the lower binding energy of 205.9 eV, which is the typical peak position for the low charge Nb^4+^.^38–40^ The presence of Nb^4+^ in the Nb_2_O_5–*x*_ NPs film also had been regarded as the reason why the film’s colour changed from white to blue after the hydrogenation (Fig. S2, ESI†).^41–43^ Fig. 2a compares the normalized O 1s core level XPS spectra of the Nb_2_O_5_ and Nb_2_O_5–*x*_ NPs samples. Significantly, the Nb_2_O_5–*x*_ NPs film shows a broader peak at around 531.68 eV than that of the Nb_2_O_5_ NPs film, indicating it has more oxygen vacancies than the Nb_2_O_5_ NPs film.^40,44–48^ This is also confirmed by the electron paramagnetic resonance (EPR) analysis. The broader peaks at *g* = 2.15 and 2.02 in Fig. 2b clearly reveal the density of oxygen vacancies in the Nb_2_O_5–*x*_ NPs film is much higher than that in the Nb_2_O_5_ NPs film.^49,50^

**Fig. 3:**
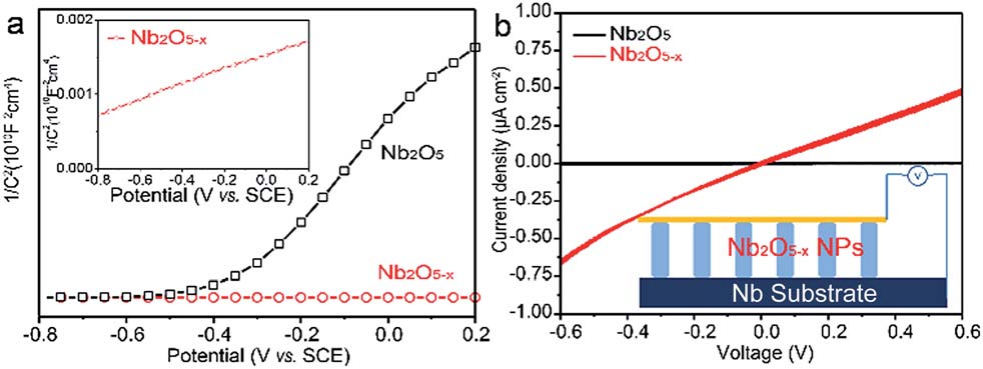
(a) Mott–Schottky plots of Nb_2_O_5_ and Nb_2_O_5–*x*_ NPs films at a frequency of 1 kHz in the dark; (b) comparison of *I*–*V* curves of the samples; the inset is the schematic of the measurement.

Electrochemical impedance measurements were carried out to verify our hypothesis that the induced oxygen vacancy can serve as a shallow donor to increase the carrier density of Nb_2_O_5_. Fig. 3a displays the Mott–Schottky plots of the electrodes at a frequency of 1 kHz in the dark, which are generated based on capacitances that derived from the electrochemical impedance. Both the Nb_2_O_5_ and Nb_2_O_5–*x*_ NPs films show positive slopes, in line with the characteristic of a n-type semiconductor. Notably, the Nb_2_O_5–*x*_ NPs film shows a substantially smaller slope for the Mott–Schottky plot than the Nb_2_O_5_ NPs film, suggesting a significantly increased donor density, based on the following equation:

3 *N*_d_ = (2/*e*_0_*εε*_0_)[d(1/*C*^2^)/d*V*]^–1^

where *N*_d_ is the donor density, *e*_0_ is the electron charge, *ε* is the dielectric constant of Nb_2_O_5_ (*ε* = 41), *ε*_0_ is the permittivity of vacuum, and *V* is the applied bias at the electrode.^41^ The carrier densities of the Nb_2_O_5_ and Nb_2_O_5–*x*_ samples are calculated to be 1.45 × 10^18^ and 3.68 × 10^23^ cm^–3^, respectively. It should be noted that here we used the electrode area instead of the surface area of the nanoporous film for the calculation, which may cause errors in determining the carrier densities. However, a qualitative comparison of the carrier densities between the Nb_2_O_5_ and Nb_2_O_5–*x*_ NP samples is reliable, since they have a similar morphology and surface area. Obviously, the Nb_2_O_5–*x*_ NPs film possesses a 5 order of magnitude improvement on the donor density compared to the Nb_2_O_5_ NPs film, which results in a significant facilitation of the charge separation in the Nb_2_O_5–*x*_ NPs film. Meanwhile, the *I*–*V* curves of the Nb_2_O_5_ and Nb_2_O_5–*x*_ NPs samples at room temperature were also collected in Fig. 3b. As expected, the electrical conductivity of the Nb_2_O_5_ sample is substantially improved after hydrogenation. All of these results validly confirm that the introduced oxygen vacancies can remarkably enhance the donor density as well as the conductivity of the Nb_2_O_5_ NPs film.

### 3.2 Performances of PEC devices based on Nb_2_O_5–*x*_ photoanodes

**Fig. 4:**
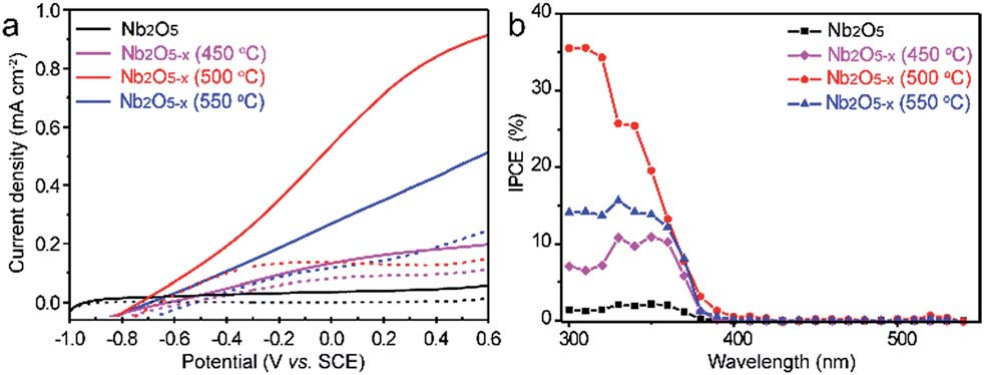
(a) *I*–*V* curves measured under 100 mW cm^–2^ simulated solar light generated by a 100 W xenon lamp coupled with an AM 1.5G filter in 1 M NaOH electrolyte; (b) IPCE spectra measured at 0.2 V *vs.* SCE in 1 M NaOH electrolyte.

The PEC performances of the Nb_2_O_5_ samples were measured in a three electrode electrochemical cell with a 1.0 M NaOH solution as the electrolyte. For optimizing the PEC performance of the Nb_2_O_5–*x*_ NPs film, the effect of the hydrogenation temperature was studied. Fig. 4a shows the linear sweep curves (LSV) of the pristine Nb_2_O_5_ and Nb_2_O_5–*x*_ NPs films obtained at different hydrogenation temperatures. The photocurrent density of the Nb_2_O_5_ NPs film is 0.05 mA cm^–2^ at 0.6 V *vs.* SCE, which is close to the value reported previously.^36^ The photocurrent densities of the Nb_2_O_5–*x*_ NPs films increase gradually with the increase of the hydrogen treatment temperature from 450 to 500 °C. The Nb_2_O_5–*x*_ (500 °C) NPs film achieves a maximum value of 0.9 mA cm^–2^ at 0.6 V *vs.* SCE, which is about a 20-fold enhancement compared to the Nb_2_O_5_ NPs film at the same potential. This photocurrent density is also dramatically higher than the values recently reported for the reported values of Nb_2_O_5_ photoanodes, such as N-doped Nb_2_O_5_ nanostructures (Table S1†).^36,51–53^ Photocurrent densities decrease gradually with the rising temperature when the annealing temperature is above 500 °C. According to the increasing donor density and conductivity of the Nb_2_O_5–*x*_ with the rising hydrogenation temperature (Fig. S4, ESI†), it can be concluded that excessive oxygen vacancies will serve as recombination centres for electrons and holes, resulting in a poor PEC performance, in agreement with other work.^54^ To quantitate the interplay between the photoactivity and light absorption, incident photon to current conversion efficiencies (IPCE) were measured for the Nb_2_O_5_ and Nb_2_O_5–*x*_ NPs photoanodes at 0.2 V *vs.* SCE (Fig. 4b). All of the Nb_2_O_5–*x*_ NPs films show significantly enhanced photoactivity over the entire UV region, and the Nb_2_O_5–*x*_ NPs film hydrogenated at 500 °C exhibits the best IPCE efficiency in the wavelength range from 300 to 380 nm. Additionally, the IPCE values decrease from 35% at 300 nm to 1% at 400 nm, and we did not observe any photoactivity in the visible light region beyond 400 nm, indicating that the observed colour change is not because of the band gap modification of Nb_2_O_5_ or the transition between the impurity states and conduction/valence band edges.^47^ Thus, we believe that the photoactivity enhancement of the Nb_2_O_5–*x*_ NPs film is because of the increased donor density and conductivity originating from the induced oxygen vacancies generated during the hydrogenation.

### 3.3 Performances of the MFC devices based on the Nb_2_O_5–*x*_ anodes

**Fig. 5:**
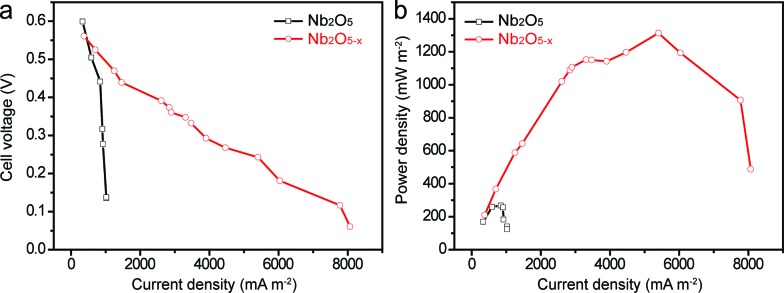
Comparisons of (a) polarization curves and (b) power outputs for Nb_2_O_5_ and Nb_2_O_5–*x*_ anodes.

To demonstrate that the as-prepared Nb_2_O_5–*x*_ NPs film is also a promising candidate as a high-performance anode material for a MFC device, a simple MFC device was assembled using the Nb_2_O_5–*x*_ NPs film hydrogenated at 500 °C as the anode and a 40 wt% Pt/C loaded carbon paper as the air cathode in the *E. coli* MFCs (denoted as Nb_2_O_5–*x*_-MFC, Experimental section). For a better comparison, the performance of the pristine Nb_2_O_5_ NPs film in the MFC device was also measured (denoted as Nb_2_O_5_-MFC). Fig. 5a and b depict the comparison of the polarization curves and power outputs tested by loading various external resistances. In comparison to the Nb_2_O_5_-MFC, the cell voltage of the Nb_2_O_5–*x*_-MFC decreased more slowly with the decreased loaded resistance and gently, indicating the superior performance of the Nb_2_O_5–*x*_ NPs anode. Furthermore, the Nb_2_O_5–*x*_-MFC achieved a remarkable maximum power density of 1196 mW m^–2^ at a current density of 4465 mA m^–2^, which is substantially higher than that of the Nb_2_O_5_-MFC (140 mW m^–2^). The distinct enhancement between the Nb_2_O_5_ and Nb_2_O_5–*x*_ MFC devices can be ascribed to the superior conductivity of the Nb_2_O_5–*x*_ NPs anode. Additionally, without *E. coli*, the Nb_2_O_5–*x*_-MFC device shows negligible cell voltage and output power density when loading the external resistances (Fig. S5†), indicating *E. coli* is very important for power production.

### 3.4 Performances of PEC–MFC hybrid devices based on Nb_2_O_5–*x*_ anodes

**Fig. 6:**
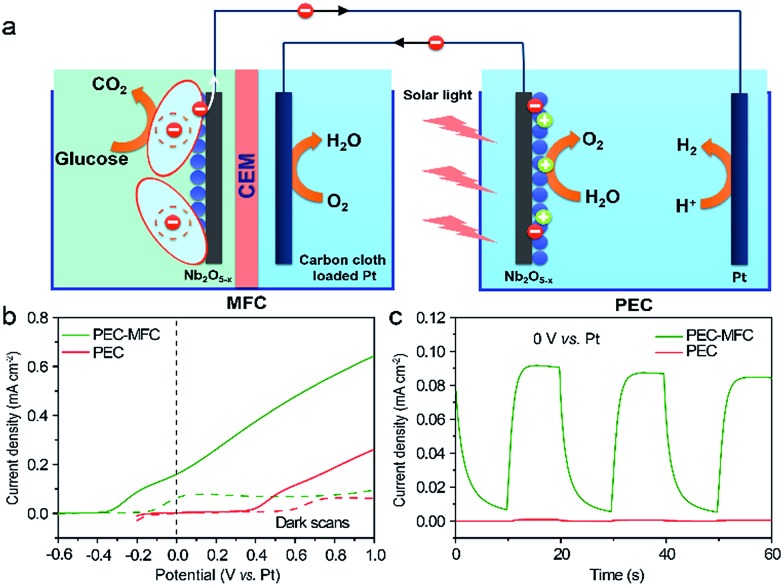
(a) Schematic configuration of a PEC–MFC device; (b) *I*–*V* curves collected from a PEC device (red line) and a PEC–MFC device (olive line) with the Nb_2_O_5–*x*_ NPs electrodes at a scan rate of 10 mV s^–1^ with/without the white light illumination; (c) *I*–*T* curves recorded for the PEC device (red line) and the PEC–MFC device (olive line) at 0 V *vs.* Pt, with light on/off cycles.

For taking full advantage of the oxygen-deficient electrodes, we assembled a PEC–MFC hybrid device to convert and store energy using the Nb_2_O_5–*x*_ NPs film as the anode in both the MFC and the PEC cell (Experimental section). The PEC properties of Nb_2_O_5–*x*_ NPs film were measured in a two-electrode electrochemical cell with/without the MFC devices in the dark and under 1 sun illumination. Fig. 6a shows the schematic configuration of the integrated PEC–MFC hybrid device. By coupling the PEC and MFC devices in series, the Nb_2_O_5–*x*_ anode in the MFC provides a biovoltage that shifted the potential of the illuminated Nb_2_O_5–*x*_ photoanode near to –0.6 V, thus, enabling water splitting to occur at zero external bias (Fig. 6b). The PEC–MFC device displays a novel photocurrent density of 0.18 mA cm^–2^ at zero bias (0 V *vs.* Pt), which is substantially larger than the one obtained from the PEC cell alone at the same potential (Fig. 6b). More importantly, gas bubbles were clearly observed to be continuously evolving on the Pt electrode under light illumination (Fig. S6 and Video S1, ESI†), indicating the generation of hydrogen. Meanwhile, the PEC device in the presence of glucose, possessing a lower performance than the hybrid device, was also conducted to emphasize the excellent energy efficiency *via* this PEC–MFC hybrid device (Fig. S7†). There is no doubt that the Nb_2_O_5–*x*_ NPs film is an outstanding material and its merits can be integrated into both a PEC cell, MFC and a hybrid device and this hybrid device is an efficient strategy to convert and store energy. The PEC–MFC device also exhibits reproducible photocurrent generation in response to light illumination (Fig. 6c), implying the hybrid device is feasible to produce hydrogen. To the best of our knowledge, this is the first demonstration of using PEC–MFC hybrid devices based on the same anode material to generate hydrogen at zero external bias.

## 4. Conclusions

In summary, we developed a facile and effective method to significantly improve the conductivity and performances of the Nb_2_O_5–*x*_ NPs films in PEC cells and MFCs through the introduction of oxygen vacancies. The Nb_2_O_5–*x*_ NPs photoanode achieved a remarkable photocurrent density of 0.9 mA cm^–2^ at 0.6 V (*vs.* SCE) in a 1 M KOH aqueous solution. Meanwhile, the Nb_2_O_5–*x*_-MFC exhibited a superior power density of 1196 mW m^–2^ when the Nb_2_O_5–*x*_ NPs film was used as an anode in the MFC cell. The oxygen vacancy plays a critical role in enhancing the effective charge transport of the electrodes, as well as increasing the conductivity. Moreover, we also demonstrated that the PEC–MFC hybrid device with the Nb_2_O_5–*x*_ NPs anodes in both devices is feasible and could produce hydrogen even at zero external bias (0 V *vs.* Pt) under illumination. To our best knowledge, it is the first report about the applications of Nb_2_O_5_ materials in an integrated PEC–MFC device to produce hydrogen without an external bias, by just using organic matter and solar light. This environmentally friendly and novel design provides a promising research direction for the future development of energy conversion and storage.
