# Supplementary material for: Solar-microbial hybrid device based on oxygen-deficient niobium pentoxide anodes for sustainable hydrogen production
Source: Chem Sci. 2015 Sep 18;6(12):6799–805. doi: 10.1039/c5sc03249k (PMC5508654; doi:10.1039/c5sc03249k)
Supplement: Supplementary file 2 [file SC-006-C5SC03249K-s002.pdf]

Electronic Supplementary Material (ESI) for Chemical Science.
This journal is © The Royal Society of Chemistry 2015

Solar-Microbial Hybrid Device based on Oxygen-deficient Niobium Pentoxide Anodes for Sustainable Hydrogen Production

Mingyang Li,[a] Xinjun He,[a] Yinxiang Zeng,[a] Meiqiong Chen,[a,b] Ziyang Zhang,[a] Hao Yang,[a] Pingping Fang,[a] Xihong Lu,*[a] and Yexiang Tong*,[a]*

[a]KLGHEI of Environment and Energy Chemistry, MOE of the Key Laboratory of Bioinorganic and Synthetic Chemistry, School of Chemistry and Chemical Engineering, Sun Yat-Sen University, Guangzhou 510275, China.

[b]Dongguan Key Laboratory of Green Energy, City college of Dongguan University of Techno logy, Wenchang Road, Dongguan, 523419 China.

Video S1. This video shows the real time hydrogen production in PEC-MFC hybrid device with/without the illumination. The PEC and MFC was combined in series. The release of gases from the Nb_2O_{5-x} NPs and bubbling can be observed starting after introducing the white light at 00:15.

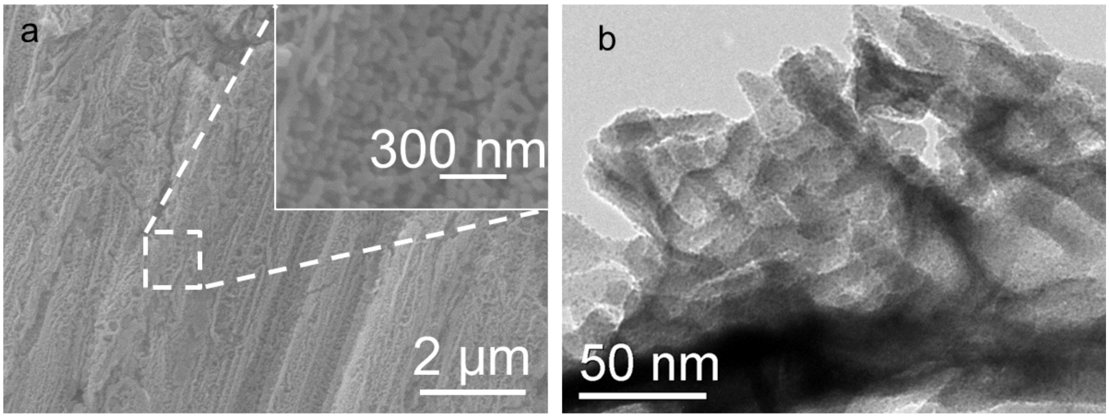

Figure S1. (a) SEM and (b) TEM images of Nb_2O_5 NPs.

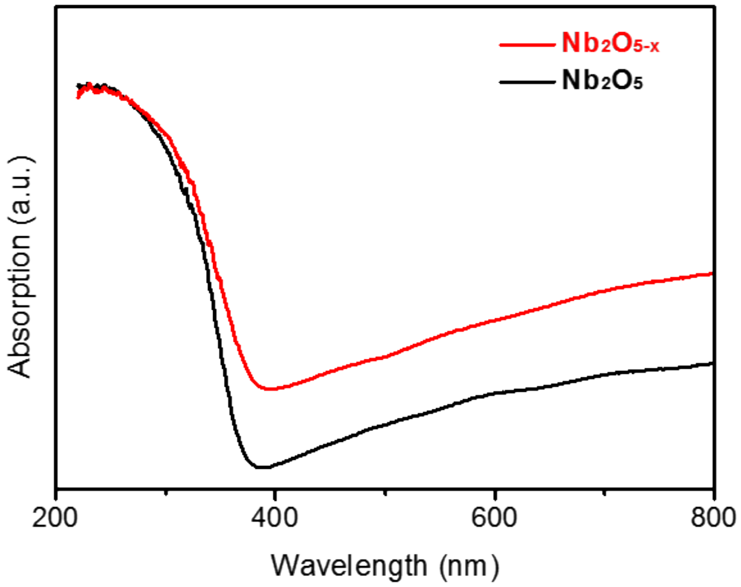

Figure S2. Absorption UV-visible spectra of Nb_2O_5 and Nb_2O_{5-x} NPs; inset: picture of Nb_2O_5 and Nb_2O_{5-x} NPs.

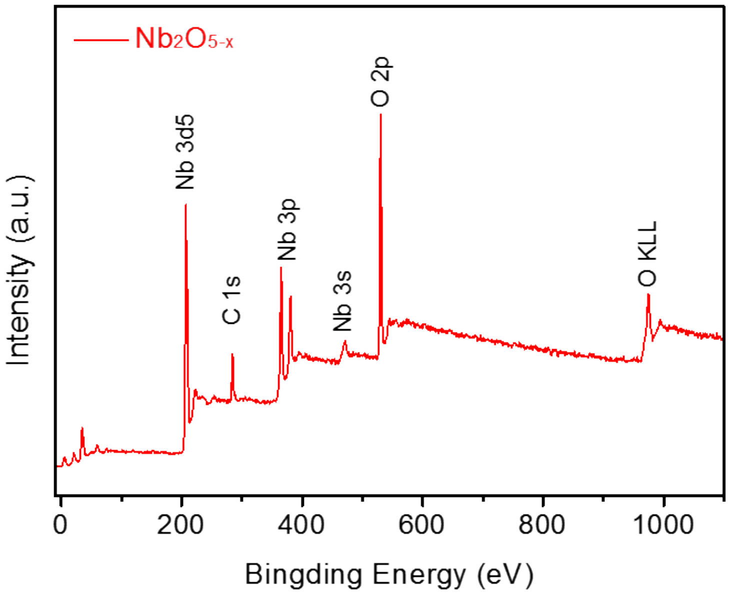

Figure S3. XPS survey spectrum collected for Nb_2O_{5-x} NPs.

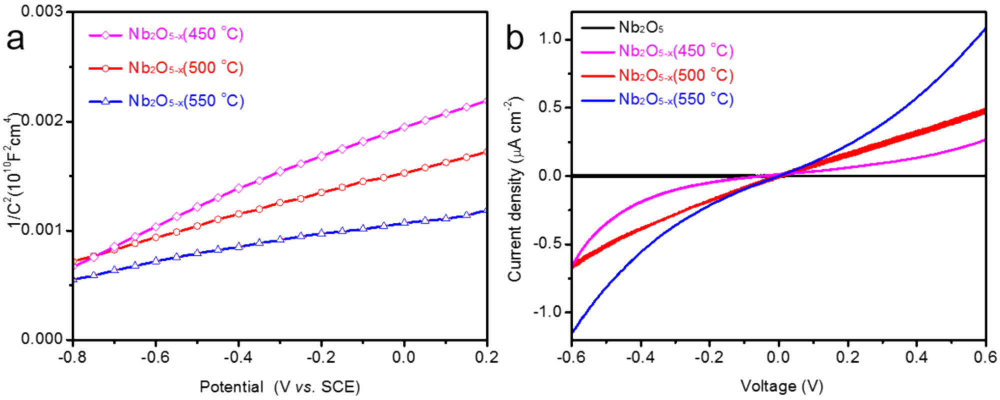

Figure S4. (a) Mott-Schottky plots of Nb_2O_{5-x} NPs treated at different temperature at a frequency of 1 kHz in the dark; (b) Comparison of *I-V* curves of the samples.

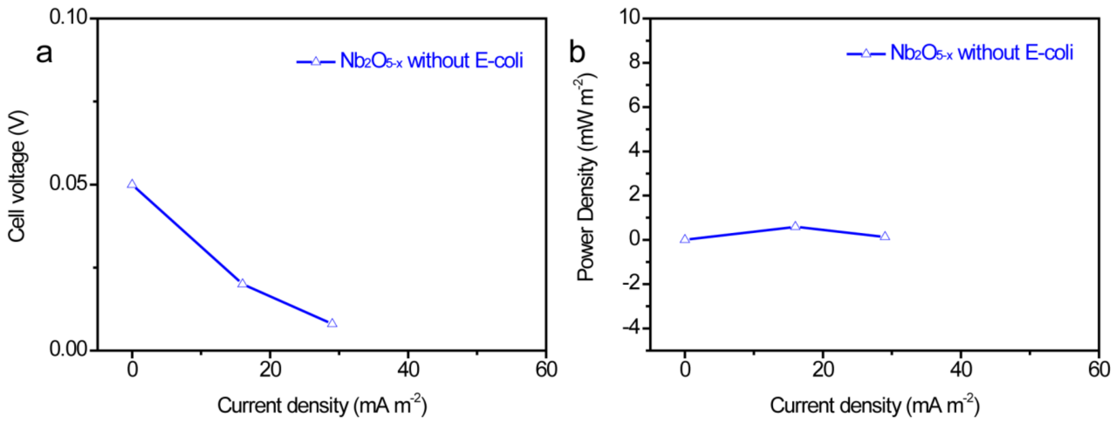

Figure S5. (a) polarization curves and (b) power outputs for Nb_2O_{5-x}-MFC without E-coli.

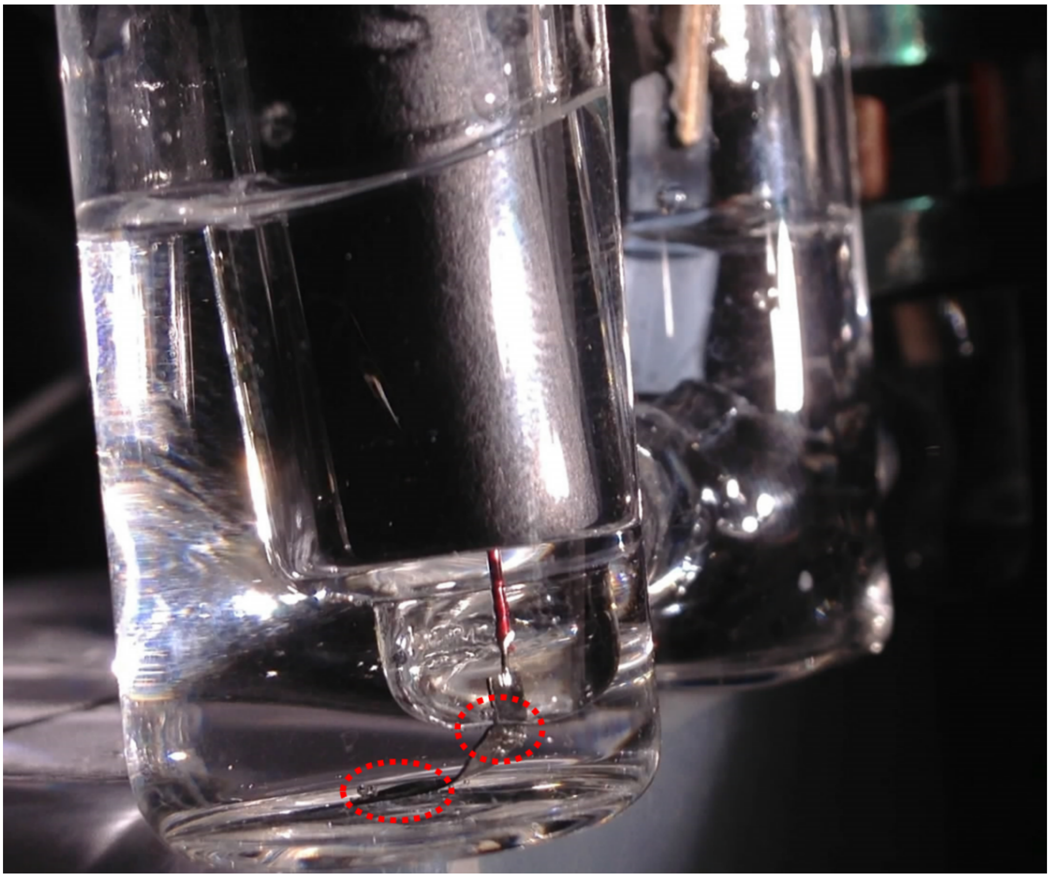

Figure S6. The red circle showing gas bubbles evolving from the Pt electrode under the illumination in the PEC-MFC hybrid device.

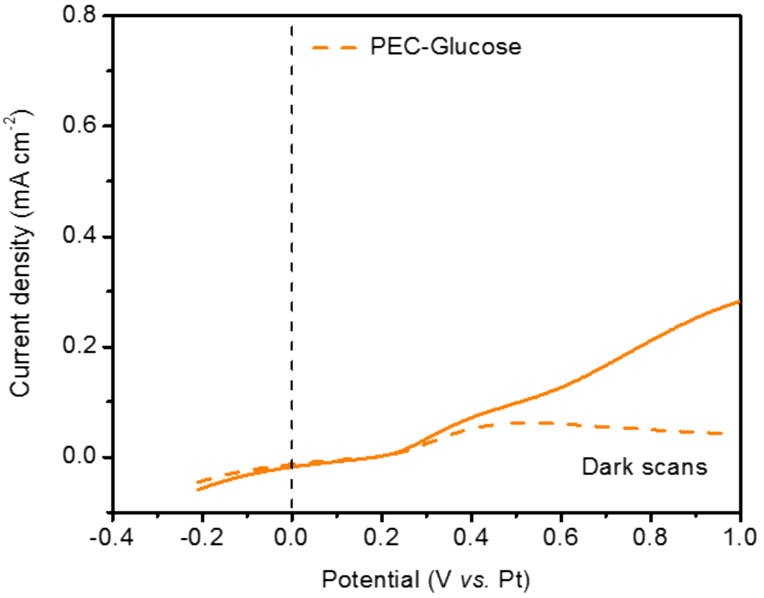

Figure S7. *I-V* curves collected from a PEC device in the presence of glucose agent (orange) with the Nb_2O_{5-x} NPs electrodes at a scan rate of 10 mV/s with/without the white light illumination.

Table S1. The summary of the photocurrent of Nb_2O_5-based electrodes.

Electrode	Electrolyte	Potential window [a](*vs.* Ag/AgCl); [b](*vs.* SCE)	Photocurrent	Illumination intensity	Ref.
N-doped Nb_2O_5	0.1 M Na_2SO_4	0 – 0.8 V [a]	0.014 mA/cm^2 at 0.8 V	N. A.	[1]
$Ag_2Nb_4O_{11}$	0.5 M Na_2SO_4	0.4 – 1.0 V [a]	0.003 mA at 1.0 V	N. A.	[2]

Bi_3NbO_7	0.5 M Na_2SO_4	0 – 1.0 V [b]	0.01 mA/cm^2 at 1.0 V	150 mW/cm^2	[3]
Fe_2O_3/Nb_2O_5	A buffer solution of pH=7 containing 0.2 M $Na_2B_4O_7$, 0.14 M H_2SO_4, and 0.3 M Na_2SO_4	0 – 1.5 V [b]	0.8 mA/cm^2 at 0.75 V	N. A.	[4]
Nb_2O_{5-x}	1.0 M NaOH	-1.0 – 0.6 V [b]	0.9 mA/cm^2 at 0.6 V	100 mW/cm^2	This work

SI References:

[1] T. Ruff, R. Hahn, M. S. Killian, H. Asoh, S. Ono, P. Schmuki, Electrochim. Acta 2012, 62, 402.

[2] H. Dong, G. Chen, J. Sun, Y. Feng, C. Li, C. Lv, Chem. Commun. 2014, 50, 6596.

[3] J. Jiang, M. Wang, Q. Chen, S. Shen, M. Li, L. Guo, RSC Adv. 2014, 4, 10542.

[4] H. Miyake, H. Kozuka, J. Phys. Chem. B 2005, 109, 17951.
